# Age and Racial Differences among PSA-Detected (AJCC Stage T1cN0M0) Prostate Cancer in the U.S.: A Population-Based Study of 70,345 Men

**DOI:** 10.3389/fonc.2013.00312

**Published:** 2013-12-23

**Authors:** Hong Zhang, Edward M. Messing, Lois B. Travis, Ollivier Hyrien, Rui Chen, Michael T. Milano, Yuhchyau Chen

**Affiliations:** ^1^Department of Radiation Oncology, University of Rochester Medical Center, Rochester, NY, USA; ^2^Department of Urology, University of Rochester Medical Center, Rochester, NY, USA; ^3^Department of Biostatistics and Computational Biology, University of Rochester Medical Center, Rochester, NY, USA

**Keywords:** prostate cancer, race, age, population-study, SEER, screen-detected

## Abstract

**Purpose**: Few studies have evaluated the risk profile of prostate-specific antigen (PSA)-detected T1cN0M0 prostate cancer, defined as tumors diagnosed by needle biopsy because of elevated PSA levels without other clinical signs of disease. However, some men with stage T1cN0M0 prostate cancer may have high-risk disease (HRD), thus experiencing inferior outcomes as predicted by a risk group stratification model.

**Methods**: We identified men diagnosed with stage T1cN0M0 prostate cancer from 2004 to 2008 reported to the surveillance, epidemiology, and end results (SEER) program. Multivariate logistic regression was used to model the probability of intermediate-risk-disease (IRD) (PSA ≥ 10 ng/ml but <20 ng/ml and/or GS 7), and high-risk-disease (HDR) (PSA ≥ 20 ng/ml, and/or GS ≥ 8), relative to low-risk disease (LRD) (PSA < 10 ng/ml and GS ≤ 6), adjusting for age, race, marital status, median household income, and area of residence.

**Results**: A total of 70,345 men with PSA-detected T1cN0M0 prostate cancer were identified. Of these, 47.6, 35.9, and 16.5% presented with low-, intermediate-, and high-risk disease, respectively. At baseline (50 years of age), risk was higher for black men than for whites for HRD (OR 3.31, 95% CI 2.85–3.84). The ORs for age (per year) for HRD relative to LRD were 1.09 (95% CI 1.09–1.10) for white men, and as 1.06 (95% CI 1.05–1.07) for black men. Further, among a subgroup of men with low PSA (<10 ng/ml) T1cN0M0 prostate cancer, risk was also higher for black man than for white men at baseline (50 years of age) (OR 2.70, 95% CI 2.09–3.48). The ORs for age (per year) for HRD relative to LRD were 1.09 (95% CI 1.09–1.10) for white men, and as 1.06 (95% CI 1.05–1.07) for black men.

**Conclusion**: A substantial proportion of men with PSA-detected prostate cancer as reported to the SEER program had HRD. Black race and older age were associated with a greater likelihood of HRD.

## Introduction

Prostate cancer is the most common malignancy in U.S. men. In 2013 alone, an estimated 241,000 new cancer cases will be diagnosed and 28,000 deaths will be attributed to prostate cancer (1). Current screening methods for prostate cancer include prostate-specific antigen (PSA) testing and digital rectal examination, although benefits of the former remain controversial (2, 3). The concern is that early detection and treatment of clinically insignificant prostate cancer may cause unnecessary side effects without added benefit.

Ever since FDA approval of PSA as a screening tool, many men have had a prostate biopsy because of an elevated PSA, despite a normal digital rectal examination. Prostate cancer diagnosed in this setting is classified as stage T1c disease based on the American Joint Commission on Cancer (AJCC) (4). Early studies have shown that stage T1c disease is heterogeneous in its pathological features. After a retrospective review of 257 patients with stage T1c prostate cancer who underwent prostatectomy and nodal dissection from 1987 to 1991, Lerner et al. (5) from the Mayo Clinic reported that 45% of patients had non-organ confined pT3 disease and 4% had node positive disease. Of 240 men with stage T1c disease who underwent radical prostatectomy at Johns Hopkins from 1994 to 1996, 28% had extracapsular extension, seminal vesicle, or lymph node involvement (6). From 1988 to 1998, 638 men with stage T1c prostate cancer underwent radical prostatectomy at Washington University, and 30% had non-organ confined pT3 or node positive disease (7). Many studies have shown that adverse pathological features such as pT3 disease (extracapsular extension and seminal vesicle involvement), high Gleason score (GS) and positive surgical margins increased the risk of disease recurrence resulting in inferior outcomes (8–17). These studies suggested that some men with stage T1c disease might have high-risk prostate cancer. However, current PSA screening protocols are not able to identify clinically significant disease in this cohort.

The purpose of this study is to provide a contemporary profile of stage T1c prostate cancer based on demographic features and a risk stratification scheme developed by D’Amico et al. (18, 19) that includes stage, GS, and PSA level. This risk stratification has been validated and widely used, including by the National Comprehensive Cancer Network (NCCN). We analyzed demographic and tumor characteristics of over 70,000 men who, according to data reported to the Surveillance, Epidemiology, and End Results (SEER) program (2004–2008), were diagnosed with prostate cancer based on an elevated PSA level and without other clinical signs of disease (stage T1cN0M0). We report the probability of high-risk prostate cancer in these patients adjusting for characteristics such as age, race, marital status, median household income, and area of residence, while taking into account pre-biopsy PSA levels. These findings may provide important information in our efforts to develop more effective prostate cancer screening tools.

## Materials and Methods

### Patient database

Men diagnosed with AJCC stage T1cN0M0 prostate adenocarcinoma at age ≥ 18 years between 2004 and 2008 and reported to the SEER 17 Registries were identified. Year 2004 was chosen as the start year, since it was February 2004 that SEER initiated collection of detailed T, N, and M staging information. “Death certificate only” and “autopsy only” cases were excluded. A total of 78,367 cases were identified with stage T1cN0M0 disease. A total of 70,345 cases had PSA or GS available for analysis.

The youngest man in the cohort with stage T1cN0M0 disease was 37 years of age. We identified 262,172 men ≥37 years of age with all stages of prostate cancer between 2004 and 2008. The SEER program provided the number of all men age ≥37 years in SEER catchment area based on 2000 U.S. population estimates.

Based on PSA levels and GS, we divided cases into the three risk groups as described by D’Amico et al. (18). Low-risk disease (LRD) was defined as PSA <10 ng/ml and GS ≤6; intermediate-risk disease (IRD) as PSA ≥10 and <20 ng/ml and/or GS 7; and high-risk disease (HRD) as PSA ≥20 ng/ml and/or GS ≥8.

Patient socioeconomic status was evaluated using “median household income” and “rural-urban continuum code” provided in the SEER program, as in prior studies (20). Median household income is an aggregate measure based on county attributes derived from the 2000 U.S. census. Rural-urban continuum code, which provides information about potential accessibility to cancer care, allows classification of counties by population size, degree of urbanization, and proximity to metropolitan areas. We grouped patients into three categories by area of residence: within metropolitan areas, adjacent to metropolitan areas, and not adjacent to metropolitan areas. Marital status at diagnosis was classified as follows: never-married (single), married, and others (widowed, divorced, and separated).

### Statistical analysis

The means of continuous variables (e.g., age and median household income) were compared between groups using *t*-tests with unequal variances. Chi-square tests were used to assess differences in categorical variables (e.g., race and gender) between groups. Multivariate logistic regression analyses were conducted to model the probability of developing IRD and HRD. The list of potential predictors to be included in the models was age, race, marital status, median household income, area of residence, and all possible first-order interactions. The analyses treated “age” as continuous variable and set 50 year of age as baseline. To select predictors significantly associated with disease risk, the data set was randomly partitioned into a training and validation data set of equal sizes (50% of the original data set each). We first ran a backward model selection procedure on the training data set to identify candidate predictors potentially associated with disease risk. Once this training step was completed, we fitted the identified model to the validation data set; predictors with *p*-values smaller than 0.05 were considered significant and included in the final models. This analysis was performed for the IRD and HRD separately. The final models were also used to model the probability of developing IRD and HRD in a subgroup analysis for patients with PSA <10 ng/ml stage T1cN0M0 prostate cancer. In all of our statistical analyses, tests were two-sided and the significance level (probability of type-1 error) was set at 0.05. Analyses were conducted using the SAS statistical package, version 9.13 (SAS Institute, Cary, NC, USA).

## Results

The age distribution of men ≥37 years of age in the SEER catchment areas are shown in Table 1. While 11.8% of men ≥37 years of age in the SEER catchment area were ≥75 years of age, 24.2% men with prostate cancer of all stages and 26.1% men with stage T1cN0M0 prostate cancer were ≥75 years of age. Among all men diagnosed with prostate cancer (*n* = 262,172) in the SEER program, 78,367 (29.9%) had stage T1cN0M0 disease. The percentage changed with age, 15.8, 24.6, 35.2, and 32.2% for age groups of 37–49, 50–64, 65–74, and ≥75 years, respectively (Table 1).

**Table 1:** **Age distribution of men in SEER catchment areas, and men with prostate cancer of all stages and stage T1cN0M0 specifically**.

Age	No. of men in SEER areas^a^	No. of men with prostate cancer		
		All stages^b^	T1cN0M0^b^	Percent (T1cN0M0/all stage)
37–49	14,983,548	1, 550	244	15.8%
50–64	12,929,972	19, 646	4, 826	24.6%
65–74	4,530,521	18, 520	6, 510	35.2%
≥75	4,329,573	12, 718	4, 093	32.2%

*^a^ Provided by SEER program using the 2000 U.S. standard population estimates*.

*^b^ Average annual number of men with all stage prostate cancer and with stage T1cN0M0 prostate cancer are defined as crude rates reported by SEER between 2004 and 2008 divided by 5*.

A total of 70,345 men with stage T1cN0M0 prostate cancer had GS and PSA information available and these men were evaluated (median age 69 years, range 37–105) (Table 2). There were 11,600 men (16.5%) with HRD. Men with HRD were significantly older (median age 72, 70, and 67 for HRD, IRD, and LRD respectively, *p* < 0.01); more likely to be black (19.4 and 15.6% for black and white respectively, *p* < 0.01); less likely to be married (16.7 and 15.5% for never-married and married respectively); and had a lower median household income (17.7, 16.6, and 15.6% for first, second, and third tertile respectively, *p* < 0.01).

**Table 2:** **Characteristics of 70,345 men with low-, intermediate-, and high-risk prostate cancer**.

	Low risk^a^	Intermediate risk^a^	High risk^a^	*p*-Value
Number of patients	33,472 (47.6%)	25,273 (35.9%)	11,600 (16.5%)	
Age, year (median, range)	67 (37–105)	70 (38–98)	72 (38–98)	<0.01
<50	720 (68.6%)	248 (23.6%)	81 (7.7%)	
50–64	12,819 (58.6%)	6,597 (30.2%)	2,449 (11.2%)	
65–74	14,418 (48.6%)	10,869 (36.6%)	4,393 (14.8%)	
≥75	5,515 (31.1%)	7,559 (42.6%)	4,677 (26.3%)	
Race				<0.01
White	26,465 (49.2%)	18,933 (35.2%)	8,366 (15.6%)	
Black	4,383 (41.5%)	4,118 (39.0%)	2,051 (19.4%)	
Other^b^	1,621 (40.8%)	1,511 (38.1%)	839 (21.1%)	
Unknown	1,003 (48.7%)	711 (34.5%)	344 (16.7%)	
Marital status				<0.01
Married	23,308 (49.1%)	16,801 (35.4%)	7,371 (15.5%)	
Never-married	3,094 (47.0%)	2,391 (36.3%)	1,099 (16.7%)	
Other^c^	3,830 (42.2%)	3,475 (38.3%)	1,772 (19.5%)	
Unknown	3,240 (45.0%)	2,606 (36.2%)	1,358 (18.9%)	
Median household income^d^				<0.01
First tertile	8,795 (46.1%)	6,919 (36.2%)	3,374 (17.7%)	
Second tertile	10,613 (47.2%)	8,149 (36.2%)	3,728 (16.6%)	
Third tertile	14,061 (48.9%)	10,205 (35.5%)	4,498 (15.6%)	
Area of residence				<0.01
Within metro	30,493 (47.8%)	22,948 (36.0%)	10,347 (16.2%)	
Adjacent to metro	1,807 (46.0%)	1,390 (35.4%)	731 (18.6%)	
Not adjacent to metro	1,138 (44.5%)	910 (35.6%)	510 (19.9%)	
Unknown	33 (47.1%)	25 (35.7%)	12 (17.1%)	
Median follow up in months (range)	26 (12–59)	25 (1–59)	25 (1–59)	
Year of diagnosis				*p* = 0.60
2004	6,176 (48.5%)	4,421 (34.7%)	2,146 (16.8%)	
2005	5,976 (47.8%)	4,425 (35.4%)	2,104 (16.8%)	
2006	7,296 (48.4%)	5,290 (35.1%)	2,489 (16.5%)	
2007	7,508 (47.2%)	5,855 (36.8%)	2,527 (15.9%)	
2008	6,516 (46.1%)	5,282 (37.4%)	2,334 (16.5%)	

*^a^ Risk groups: low risk – PSA <10 ng/ml and GS ≤6, intermediate risk – 10 ng/ml ≤ PSA <20 ng/ml and/or GS = 7, high risk – PSA >20 ng/ml, and/or GS ≥8 as defined by D’Amico (19)*.

*^b^ Other: American Indian, Alaska Native, Asian, and Pacific Islander*.

*^c^ Other: widowed, divorced, and separated*.

*^d^ First tertile, <$ 42,190; second tertile, $ 42,191–49,820; third tertile, ≥$ 49,821*.

Based on age and PSA level, Table 3 shows the distribution of GS among 70,345 men with stage T1cN0M0 disease. For example, a 72-year-old man with a PSA of 8 ng/ml stage T1cN0M0 prostate cancer has a 7.2% chance of having GS ≥8 HRD. For an 82-year-old man with the same PSA level, the chance of HRD is 16.5%.

**Table 3:** **Distribution of low-, intermediate-, and high- risk T1cN0M0 prostate cancer according to age and PSA**.

Age	PSA (ng/ml)														
	<4			4–9.9			10–14.9			15–19.9			≥20		
<50	195 (84.8%)	33 (14.4%)	2 (0.9%)	525 (78.9%)	124 (18.6%)	15 (2.3%)	40 (49.4%)	34 (42.0%)	7 (8.6%)	10 (55.6%)	7 (38.9%)	1 (5.6%)	27 (48.2%)	16 (28.6%)	13 (23.2%)
50–54	372 (81.9%)	75 (16.5%)	7 (1.5%)	1,541 (73.5%)	496 (23.7%)	58 (2.8%)	165 (58.7%)	92 (32.7%)	23 (8.2%)	45 (51.1%)	32 (36.4%)	10 (11.4%)	86 (37.7%)	100 (43.9%)	41 (18.0%)
55–59	647 (81.0%)	133 (16.6%)	18 (2.3%)	3,771 (77.8%)	1,191 (23.0%)	221 (4.3%)	392 (58.5%)	219 (32.7%)	59 (8.8%)	100 (48.8%)	72 (35.1%)	33 (16.1%)	204 (44.2%)	161 (34.8%)	97 (21.0%)
60–64	765 (75.4%)	198 (19.5%)	51 (5.0%)	5,723 (70.0%)	2,045 (25.0%)	411 (5.0%)	638 (59.6%)	344 (32.1%)	89 (8.3%)	201 (47.6%)	159 (37.7%)	62 (14.7%)	321 (44.7%)	253 (35.2%)	144 (10.1%)
65–69	806 (71.6%)	256 (22.7%)	64 (5.7%)	6,829 (66.3%)	2,868 (27.8%)	609 (5.9%)	893 (54.2%)	549 (33.3%)	206 (12.5%)	261 (49.7%)	185 (35.2%)	79 (15.0%)	389 (36.9%)	427 (40.6%)	237 (22.5%)
70–74	651 (66.6%)	246 (25.2%)	80 (8.2%)	6,136 (60.6%)	3,258 (32.2%)	733 (7.2%)	1,076 (54.2%)	663 (33.4%)	247 (12.4%)	352 (48.2%)	262 (35.8%)	117 (16.0%)	431 (35.8%)	452 (37.5%)	322 (26.7%)
75–79	365 (53.2%)	235 (34.3%)	86 (12.5%)	3,769 (53.9%)	2,493 (35.6%)	736 (10.5%)	908 (46.3%)	708 (36.1%)	344 (17.6%)	274 (42.9%)	222 (34.7%)	143 (22.4%)	377 (33.4%)	441 (39.1%)	310 (27.5%)
≥80	335 (46.1%)	281 (38.7%)	110 (15.2%)	1,046 (44.3%)	925 (39.2%)	389 (16.5%)	481 (35.2%)	558 (40.8%)	328 (24.0%)	205 (31.4%)	269 (41.2%)	179 (27.4%)	248 (20.1%)	512 (41.5%)	474 (38.4%)
	LRD^a^	IRD^b^	HRD^c^	LRD^a^	IRD^b^	HRD^c^	IRD^b^	IRD^b^	HRD^c^	IRD^b^	IRD^b^	HRD^c^	HRD^c^	HRD^c^	HRD^c^
	*****≤***6**	**7**	*****≥***8**	*****≤***6**	**7**	*****≥***8**	*****≤***6**	**7**	*****≥***8**	*****≤***6**	**7**	*****≥***8**	*****≤***6**	**7**	*****≥***8**
	Gleason score														

*^a^ Low-risk disease – PSA <10 ng/ml and GS ≤6 T1cN0M0 prostate cancer*.

*^b^ Intermediate-risk disease – 10 ng/ml ≤ PSA <20 ng/ml and/or GS = 7 T1cN0M0 prostate cancer*.

*^c^ High-risk disease – PSA >20 ng/ml and/or GS ≥8 T1cN0M0 prostate cancer*.

**Figure 1:**
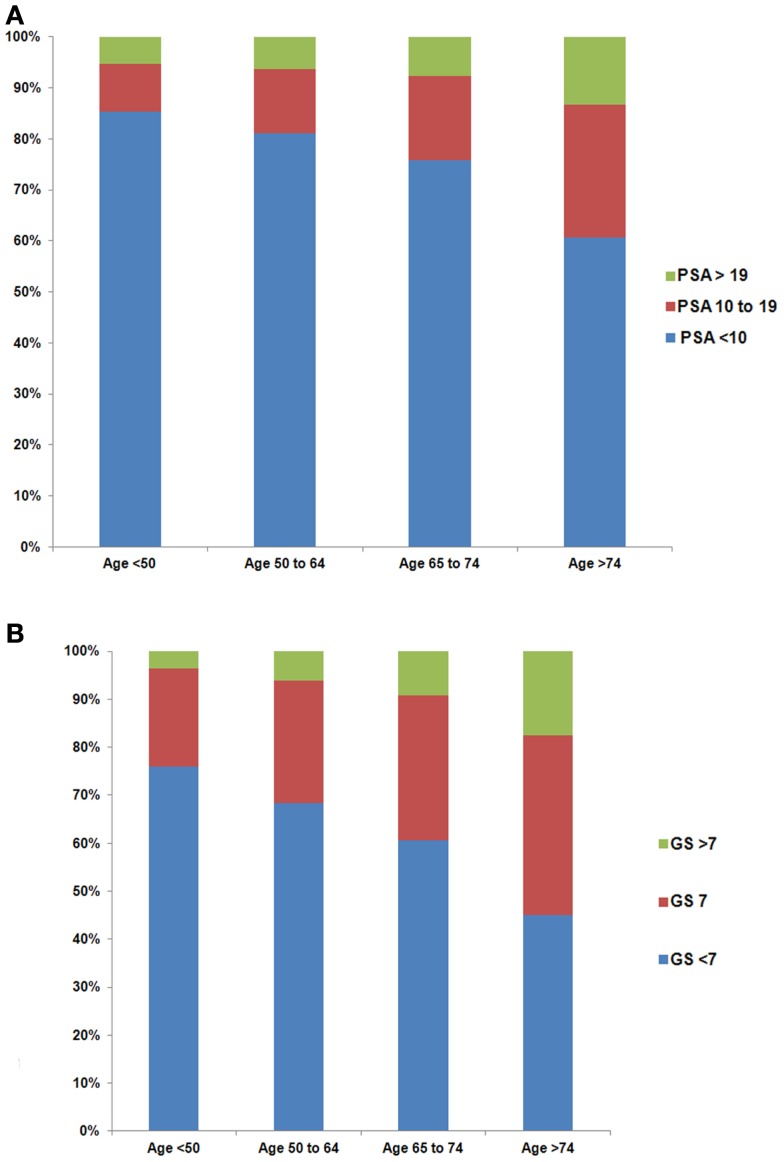
**Distribution of PSA (A) and Gleason Score (GS) (B) by age group among men with stage T1cN0M0 prostate cancer**. The difference in the distribution of PSA (*p* < 0.01) and GS (*p* < 0.01) among age groups was evaluated with the two-sided Chi-square test.

**Figure 2:**
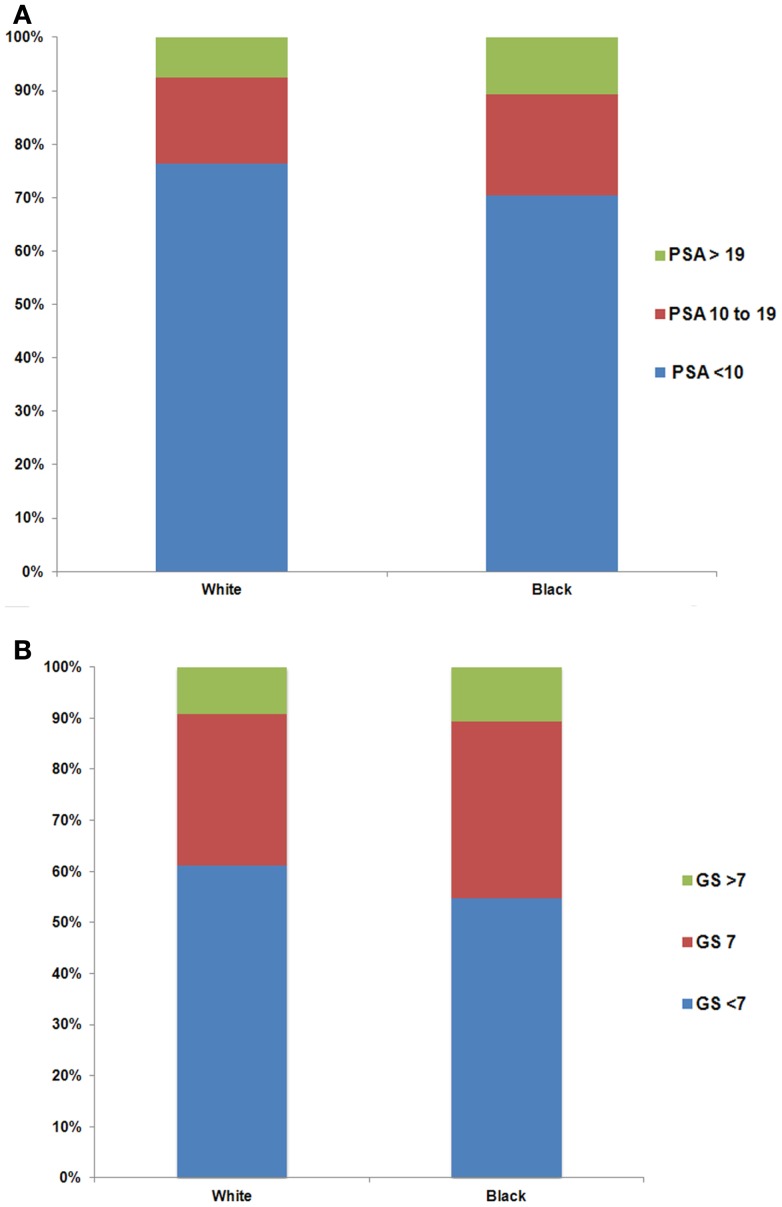
**Distribution of PSA (A) and Gleason Score (GS) (B) by white and black race among men with stage T1cN0M0 prostate cancer**. The difference in the distribution of PSA (*p* < 0.01) and GS (*p* < 0.01) between white and black men was evaluated with the two-sided Chi-square test.

Older men were more likely to have higher PSA levels at the time of diagnosis (Figure 1A, *p* < 0.01). The percentage of men with PSA ≥20 ng/ml stage T1cN0M0 prostate cancer increased with age (5.3, 6.4, 7.6, and 13.3% for age groups 37–49, 50–64, 65–74, and ≥75, respectively). Furthermore, the percentage of men with higher GS increased with age (Figure 1B, *p* < 0.01). There were 3.6, 6.1, 9.1, and 17.5% men with GS ≥8 stage T1cN0M0 prostate cancer for age groups 37–49, 50–64, 65–74, and ≥75, respectively. Black men were more likely to have higher PSA levels than white men at diagnosis (Figure 2A, *p* < 0.01). For example, 10.6% of black men and 7.5% of white men had PSA of ≥20 ng/ml. There was a small but significant difference in GS profile between black and white men (Figure 2B, *p* < 0.01).

Multivariate logistic regression analyses identified a significant interaction between age and race, indicating that risk increased faster with age among white men compared to blacks for both HRD and IRD relative to LRD (Table 4). At baseline (50 years of age), risk was higher among black men than among whites for HRD and IRD (OR 3.31 and 2.02, 95% CI 2.85–3.84, and 1.81–2.25, respectively). The ORs for age (per year) for HRD and IRD relative to LRD were estimated as 1.09 and 1.06 respectively (95% CI 1.09–1.10, 1.05–1.06) for white men, and as 1.06 and 1.04 respectively (95% CI 1.05–1.07, 1.03–1.04) for black men. Compared to baseline (50 years of age), the ORs at 75 years of age for HRD and IRD relative to LRD were estimated as 9.25 (95% CI 8.45–10.13) and 3.97 (95% CI 3.72–4.24) respectively for white men, and as 4.24 (95% CI 3.56–5.04) and 2.58 (95% CI 2.24–2.96) respectively for black men. Men in the “never-married” category compared with “married” men were more likely to have HRD and IRD (OR 1.35 and 1.22, 95% CI 1.25–1.46 and 1.15–1.29, respectively), but no significant association was observed between median household income and disease risk.

**Table 4:** **Multivariate logistic regression analyses modeling the probabilities of patients with intermediate-risk disease vs. low-risk disease, and of patients with high-risk diseases vs. low-risk disease respectively**.

	Intermediate vs. low risk^a^			High vs. low risk^a^		
	Odds ratio	95% CI^b^	*p*-Value	Odds ratio	95% CI^b^	*p*-Value
Age (per year) × race
White	1.06	(1.05, 1.06)	<0.01	1.09	(1.09, 1.10)	<0.01
Black	1.04	(1.03, 1.04)	<0.01	1.06	(1.05, 1.07)	<0.01
Other^c^	1.06	(1.04, 1.07)	0.86	1.08	(1.07, 1.11)	0.27
Race			<0.01			<0.01
White	–	–	–	–	–	–
Black	2.02	(1.81, 2.25)	<0.01	3.31	(2.85, 3.84)	<0.01
Other^c^	1.30	(1.06, 1.60)	0.01	1.58	(1.40, 2.46)	<0.01
Marital status			<0.01			<0.01
Married	–	–	–	–	–	–
Never-married	1.22	(1.15, 1.29)	<0.01	1.35	(1.25, 1.46)	<0.01
Others^d^	1.19	(1.13, 1.26)	<0.01	1.34	(1.25, 1.43)	<0.01
Median household income^e^			0.43			0.42
First tertile	–	–	–	–	–	–
Second tertile	0.98	(0.94, 1.03)	0.50	0.98	(0.92, 1.05)	0.56
Third tertile	0.97	(0.92, 1.02)	0.20	0.96	(0.90, 1.02)	0.20
Area of residence			0.66			<0.01
Within metro	–	–	–	–	–	–
Adjacent to metro	1.00	(0.92, 1.09)	0.93	1.11	(1.00, 1.23)	0.06
Not adjacent to metro	1.05	(0.95, 1.16)	0.37	1.29	(1.13, 1.46)	<0.01

*^a^ Risk groups: low risk – PSA <10 ng/ml and GS ≤6, intermediate risk – 10 ng/ml ≤ PSA <20 ng/ml and/or GS = 7, high risk – PSA ≥20 ng/ml and/or GS ≥8*.

*^b^ Cl, confidence interval*.

*^c^ Others: American Indian, Alaska Native, Asian, and Pacific Islander*.

*^d^ Other: widowed, divorced, and separated*.

*^e^ First tertile, <$ 42,190; second tertile, $ 42, 191–49,820; third tertile, ≥$ 49,821*.

As one may argue that the elevated PSA levels in the elderly patients with HRD may be a function of lead-time detection of younger individuals with lower PSA, we also analyzed age and racial effect in a subgroup of men with PSA <10 ng/ml stage T1cN0M0 prostate cancer (Table 5). We found that in this group of men with low PSA (<10 ng/ml), again, older men were more likely to have GS ≥8 HRD (12.3, 6.6, 4.3, and 1.9% for age groups ≥75, 65–74, 50–64, and 37–49 respectively). Black men were more likely to have GS ≥8 HRD (7.6 and 6.7% for black and white men respectively). Multivariate logistic regression analyses for this cohort of men showed that risk increased faster with age among white men compared to black men for both HRD and IRD relative to LRD (Table 6). At baseline (50 years of age), risk was higher among black men than among whites for HRD and IRD (OR 2.70 and 1.94, 95% CI 2.09–3.48 and 1.70–2.20, respectively). The OR for age (per year) for patients with GS ≥8 HRD (relative to LRD) were 1.09 (95% CI 1.09–1.10) for white men and 1.06 (95% CI 1.05–1.07) for black men. Compared to baseline 50 years of age, the ORs for 75-year-old men for HRD and IRD relative to LRD were estimated as 8.51 (95% CI 7.36–9.86) and 3.25 (95% CI 3.01–4.51) respectively for white men, and as 4.09 (95% CI 3.05–5.50) and 2.09 (95% CI 1.78–2.46) respectively for black men.

**Table 5:** **Characteristics of 51,919 men with PSA <10 ng/ml stage T1cN0M0 prostate cancer**.

	Low risk^a^	Intermediate risk^a^	High risk^a^	*p*-Value
Number of patients	33472 (64.5%)	14857 (28.6%)	3590 (6.9%)	
Age, year (median, range)	67 (37–105)	70 (38–95)	72 (44–95)	<0.01
<50	720 (80.5%)	157 (17.6%)	17 (1.9%)	
50–64	12819 (72.3%)	4138 (23.3%)	766 (4.3%)	
65–74	14418 (64.0%)	6628 (29.4%)	1486 (6.6%)	
≥75	5515 (51.2%)	3934 (36.5%)	1321 (12.3%)	
Race				<0.01
White	26465 (65.4%)	11318 (28.0%)	2697 (6.7%)	
Black	4383 (59.9%)	2380 (32.5%)	560 (7.6%)	
Other^b^	1621 (61.2%)	778 (29.4%)	250 (9.4%)	
Unknown	1003 (68.4%)	381 (26.0%)	83 (5.7%)	
Marital status				<0.01
Married	23,308 (65.0%)	10,117 (28.2%)	2,444 (6.8%)	
Never-married	3,094 (65.9%)	1,302 (27.7%)	297 (6.3%)	
Other^c^	3,830 (61.5%)	1,891 (30.4%)	505 (8.1%)	
Unknown	3,240 (62.9%)	1,547 (30.4%)	344 (6.8%)	
Median household income^d^				<0.01
First tertile	8,795 (63.9%)	3,972 (28.9%)	996 (7.2%)	
Second tertile	10,613 (65.1%)	4,583 (28.1%)	1,108 (6.8%)	
Third tertile	14,061 (64.4%)	6,299 (28.8%)	1,486 (6.8%)	
Area of residence				<0.01
Within metro	30,493 (64.4%)	13,588 (28.7%)	3,263 (6.9%)	
Adjacent to metro	1,807 (64.5%)	790 (28.2%)	204 (7.3%)	
Not adjacent to metro	1,138 (65.7%)	471 (27.2%)	122 (7.0%)	
Year of diagnosis				
2004	6,176 (68.2%)	2,317 (25.6%)	563 (6.2%)	
2005	5,976 (66.3%)	2,421 (26.9%)	616 (6.8%)	
2006	7,296 (65.3%)	3,098 (27.7%)	779 (7.0%)	
2007	7,508 (62.8%)	3,597 (30.1%)	845 (7.1%)	
2008	6,516 (60.7%)	3,424 (31.9%)	787 (7.3%)	

*^a^ Risk groups: low risk – PSA <10 ng/ml and GS ≤6, intermediate risk – PSA <10 ng/ml and GS = 7, high risk – PSA <10 ng/ml and GS ≥8*.

*^b^ Other: American Indian, Alaska Native, Asian, and Pacific Islander*.

*^c^ Other: widowed, divorced, and separated*.

*^d^ First tertile, <$ 42,190; second tertile, $ 42,191–49,820; third tertile, ≥$ 49,821*.

**Table 6:** **Multivariate logistic regression analyses modeling the probabilities of patients with PSA <10 ng/ml intermediate-risk disease vs. low-risk disease, and of patients with PSA <10 ng/ml high-risk diseases vs. low-risk disease respectively**.

	Intermediate vs. low risk^a^			High vs. low risk^a^		
	Odds ratio	95% CI^b^	*p*-Value	Odds ratio	95% CI^b^	*p*-Value
Age (per year) × race
White	1.05	(1.04, 1.05)	<0.01	1.09	(1.09, 1.10)	<0.01
Black	1.04	(1.04, 1.05)	<0.01	1.06	(1.05, 1.07)	<0.01
Other^c^	1.04	(1.03, 1.06)	0.26	1.08	(1.06, 1.10)	0.26
Race			<0.01			<0.01
White	–	–	–	–	–	–
Black	1.94	(1.70, 2.20)	<0.01	2.70	(2.09, 3.48)	<0.01
Other^c^	1.27	(0.99, 1.63)	0.06	1.85	(1.17, 2.91)	<0.01
Marital status			<0.01			<0.01
Married	–	–	–	–	–	–
Never-married	1.07	(0.99, 1.15)	0.07	1.11	(0.97, 1.27)	0.12
Others^d^	1.09	(1.03, 1.16)	<0.01	1.18	(1.06, 1.31)	<0.01
Median household income^e^			0.09			0.45
First tertile	–	–	–	–	–	–
Second tertile	0.95	(0.90, 1.01)	0.12	0.95	(0.85, 1.05)	0.29
Third tertile	1.01	(0.95, 1.07)	0.82	0.99	(0.90, 1.10)	0.90
Area of residence			0.19			0.84
Within metro	–	–	–	–	–	–
Adjacent to metro	0.95	(0.86, 1.04)	0.27	0.98	(0.83, 1.67)	0.84
Not adjacent to metro	0.90	(0.80, 1.02)	0.11	0.94	(0.75, 1.17)	0.57

*^a^ Risk groups: low risk – PSA <10 ng/ml and GS ≤6, intermediate risk – PSA <10 ng/ml and GS = 7, high risk – PSA <10 ng/ml and GS ≥8*.

*^b^ Cl, confidence interval*.

*^c^ Others: American Indian, Alaska Native, Asian, and Pacific Islander*.

*^d^ Other: widowed, divorced, and separated*.

*^e^ First tertile, <$ 42,190; second tertile, $ 42,191–49,820; third tertile, ≥$ 49,821*.

## Discussion

To our knowledge, this is the largest population-based study focused only on PSA-detected (stage T1cN0M0) prostate cancer in the U.S. in the contemporary era of widespread PSA testing. A significant number of men (16.5%) in this cohort had HRD. We found that men of older age and black race were more likely to have HRD than younger and white men.

According to the U.S. census, 4.6% of the total U.S. male population in 2010 was ≥75 years of age respectively (21). However, 40.3% of men with HRD were ≥75 years of age. Further, 36.8% of men with PSA <10 ng/ml and GS ≥8 stage T1cN0M0 prostate cancer were ≥75 years of age. Our finding that older men were more likely to have HRD, albeit limited to PSA-detected stage T1cN0M0 disease in this survey, is consistent with recently published studies (22, 23). After evaluating all prostate cancer cases reported to the SEER program from 1998 to 2007, Scosyrev et al. (22) reported that men ≥75 years of age were more likely to present with either metastatic or locally advanced disease, and experienced the highest prostate cancer-specific mortality. Bechis et al. (23) reviewed the database of the Cancer of the Prostate Strategic Urologic Research Endeavor (CaPSURE) and reported that 26% of men age ≥75 had HRD based on the Cancer of the Prostate Risk Assessment (CAPRA) score. In addition to clinical stage, PSA, and GS, CAPRA scores take into consideration patient age and percentage of biopsy cores involved with prostate cancer.

The specific indications for PSA testing and prostate biopsy could not be confirmed in our series, given the limitations of the SEER database. Nonetheless, all cases reported to the SEER program were diagnosed by a needle biopsy because of an elevated PSA level with no other clinical signs of disease (AJCC T1cN0M0) (4). The higher proportion of older men with HRD reported here cannot be explained by potential bias that older men with inherently higher PSA levels were more likely to have biopsy. Even among men with PSA <10 ng/ml stage T1cN0M0 disease, older men had significant risk of HRD. One of many possible explanations is that older men may harbor aggressive disease that is not reflected by PSA level. After pathological review of 211 autopsied prostate glands from deceased men with no known prostate cancer at the time of death, Delongchamps et al. (24) reported older men had significantly larger tumors, higher GS, and were more likely to have extraprostatic extension or microscopic invasion of bladder neck (4). After a prospective review of 268 men with stage T1c prostate cancer who underwent radical prostatectomy at seven U.S. medical centers, Southwick et al. (25) noted age was one of the significant predictors of unfavorable pathological outcome, including extracapsular extension, seminal vesicle invasion, invasion of bladder neck/rectum, and lymph node involvement. Further study is needed to evaluate many confounding factors on observed age effect.

African Americans have the highest prostate cancer burden and mortality of any racial group (1). Our study showed that in the cohort of men with PSA screen-detected stage T1cN0M0 disease, African Americans had a higher likelihood of harboring HRD than whites, including cohort of men with PSA <10 ng/ml. Many socioeconomic and intrinsic factors may contribute to such a racial difference. But even among U.S. service men with equal access to care, racial difference in prostate cancer risk remains (26). In an early study among men with non-palpable prostate cancer (clinical stage T1c disease) who underwent prostatectomy, Sanchez-Ortiz et al. reported that African American men had higher GS and greater tumor volume (27). Therefore, prostate cancer in black men may be biologically different from whites. In fact, several genetic and biological mechanisms have been identified that may contribute to the aggressiveness of prostate cancer in African American men (28–32).

Our study was not designed to address the question of whether earlier detection would improve survival of men with HRD. Also to our knowledge, there have been no reported randomized studies that evaluate the outcome of early intervention vs. active surveillance for men found to have stage T1cN0M0 prostate cancer. There are two UK-based ongoing prostate cancer trials: the CAP (Comparison Arm for ProtecT) and ProtecT (Prostate Testing for Cancer and Treatment) trials (33) that may help address issues of screening and treatment, but the results will not be available until 2016. Based on results from many published studies (34–36) including stage T1cN0M0 disease, it is conceivable that a subgroup of men may indeed benefit from early detection and treatment. The challenge at this time is to distinguish these patients from many men with clinically non-consequential disease.

Our study has several weaknesses due to the retrospective design and inherent deficiencies of data reported to the SEER program. We had no way to independently verify staging accuracy. SEER did not provide detailed information about biopsy templates/schemes. We were not able to analyze patient outcomes due to the short follow up in the cohort. Further, there was no information about patients’ performance status, medical co-morbidities, voiding symptoms, or family history of prostate cancer in the SEER database; these factors might have influenced screening decisions. Eight percent (5,605/70,345) of men in this study had a PSA level <4 ng/ml. It is unclear why these men proceeded to biopsy, though likely explanations may include patient preference, family history of prostate cancer, or incorrect staging.

We used a risk stratification scheme developed by D’Amico et al. (18, 19) that has been validated and widely used. In addition to clinical stage, PSA, and GS, there are many other factors that may influence prostate cancer outcome. These include primary and secondary GS, tertiary GS (37–41), percentage of positive biopsies (42–47), or presence of perineural invasion in the biopsy specimen (48). The SEER database does not provide such important information; therefore we limited our risk estimation based on clinical stage (in this case, stage T1cN0M0), PSA, and GS.

Despite the above limitations, our large population-based study shows that a substantial proportion of men with PSA-detected stage T1cN0M0 prostate cancer may have HRD in the contemporary era. Older and black men were more likely to have HRD than younger and white men. Analytic studies with independently verified staging information are needed to confirm these findings, and examine clinical outcomes in these men, especially those of older age and of black race.

## Author Contributions

Conception and design: Hong Zhang, Edward M. Messing, Lois B. Travis, and Yuhchyau Chen; Collection and assembly of data: Hong Zhang; Data analysis and interpretation: all authors; Manuscript writing: all authors; Final approval of manuscript: all authors.

## Conflict of Interest Statement

The authors declare that the research was conducted in the absence of any commercial or financial relationships that could be construed as a potential conflict of interest.

## References

[1] SEER Stat Facts Sheet: Cancer of the Prostate. Available from: www.seer.cancer.gov/statfacts/html/prost.html

[2] Peres J New PSA guidelines discourage overscreening. J Natl Cancer Inst (2012) 104:8–9 10.1093/jnci/djr539 22173585

[3] Schröder FH Hugosson J Roobol MJ Tammela TL Ciatto S Nelen V Screening and prostate-cancer mortality in a randomized European study. N Engl J Med (2009) 360:1320–8 10.1056/NEJMoa0810084 19297566

[4] Edge SB Byrd DR Compton CC AJCC Cancer Staging Manual, Seventh Edition. New York: Springer Science+Business Media (2009).

[5] Lerner SE Seay TM Blute ML Bergstralh EJ Barrett D Zincke H Prostate specific antigen detected prostate cancer (clinical stage T1c): an interim analysis. J Urol (1996) 155:821–6 10.1097/00005392-199603000-00005 8583584

[6] Carter HB Sauvageot J Walsh PC Epstein JI Prospective evaluation of men with stage T1C adenocarcinoma of the prostate. J Urol (1997) 157:2206–9 10.1016/S0022-5347(01)64719-0 9146616 PMC3461836

[7] Ramos CG Carvalhal GF Smith DS Mager DE Catalona WJ Clinical and pathological characteristics, and recurrence rates of stage T1c versus T2a or T2b prostate cancer. J Urol (1999) 161:1525–9 10.1016/S0022-5347(05)68944-6 10210388

[8] Stephenson AJ Kattan MW Eastham JA Dotan ZA Bianco FJ Jr Lilja H Defining biochemical recurrence of prostate cancer after radical prostatectomy: a proposal for a standardized definition. J Clin Oncol (2006) 24:3973–8 10.1200/JCO.2005.04.0756 16921049

[9] Swindle P Eastham JA Ohori M Kattan MW Wheeler T Maru N Do margins matter? The prognostic significance of positive surgical margins in radical prostatectomy specimens. J Urol (2005) 174:903–7 10.1097/01.ju.0000169475.00949.78 16093984

[10] Kupelian PA Katcher J Levin HS Klein EA Stage T1-2 prostate cancer: a multivariate analysis of factors affecting biochemical and clinical failures after radical prostatectomy. Int J Radiat Oncol Biol Phys (1997) 37:1043–52 10.1016/S0360-3016(96)00590-1 9169811

[11] Epstein JI Pizov G Walsh PC Correlation of pathologic findings with progression after radical retropubic prostatectomy. Cancer (1993) 71:3582–93 10.1002/1097-0142(19930601)71:11<3582::AID-CNCR2820711120>3.0.CO;2-Y 7683970

[12] Zietman AL Edelstein RA Coen JJ Babayan RK Krane RJ Radical prostatectomy for adenocarcinoma of the prostate: the influence of preoperative and pathologic findings on biochemical disease-free outcome. Urology (1994) 43:828–33 10.1016/0090-4295(94)90144-9 7515206

[13] Lee HM Solan MJ Lupinacci P Gomella LG Valicenti RK Long-term outcome of patients with prostate cancer and pathologic seminal vesicle invasion (pT3b): effect of adjuvant radiotherapy. Urology (2004) 64:84–9 10.1016/j.urology.2004.02.004 15245941

[14] Ohori M Wheeler TM Kattan MW Goto Y Scardino PT Prognostic significance of positive surgical margins in radical prostatectomy specimens. J Urol (1995) 154:1818–24 10.1097/00005392-199511000-00063 7563355

[15] Lowe BA Lieberman SF Disease recurrence and progression in untreated pathologic stage T3 prostate cancer: selecting the patient for adjuvant therapy. J Urol (1997) 158:1452–6 10.1097/00005392-199710000-00036 9302141

[16] Pound CR Partin AW Eisenberger MA Chan DW Pearson JD Walsh PC Natural history of progression after PSA elevation following radical prostatectomy. JAMA (1999) 281:1591–7 10.1001/jama.281.17.1591 10235151

[17] Catalona WJ Smith DS 5-year tumor recurrence rates after anatomical radical retropubic prostatectomy for prostate cancer. J Urol (1994) 152(5 Pt 2):1837–42 7523731 10.1016/s0022-5347(17)32397-2

[18] D’Amico AV Whittington R Malkowicz SB Cote K Loffredo M Schultz D Biochemical outcome after radical prostatectomy or external beam radiation therapy for patients with clinically localized prostate carcinoma in the prostate specific antigen era. Cancer (2002) 95:281–6 10.1002/cncr.10657 12124827

[19] D’Amico AV Whittington R Malkowicz SB Schultz D Blank K Broderick GA Biochemical outcome after radical prostatectomy, external beam radiation therapy, or interstitial radiation therapy for clinically localized prostate cancer. JAMA (1998) 280:969–74 10.1001/jama.280.11.969 9749478

[20] Zhang H Travis LB Chen R Hyrien O Milano MT Newlands SD Impact of radiotherapy on laryngeal cancer survival: a population-based study of 13,808 US patients. Cancer (2012) 118:1276–87 10.1002/cncr.26357 21773970

[21] Werner CA The Older Population 2010: 2010 Census Briefs. U.S. Census Bureau, U.S. Department of Commerce, Economics and Statistics Administration. (2011). Available from: http://www.census.gov/prod/cen2010/briefs/c2010br-09.pdf

[22] Scosyrev E Messing EM Mohile S Golijanin D Wu G Prostate cancer in the elderly: frequency of advanced disease at presentation and disease-specific mortality. Cancer (2012) 118:3062–70 10.1002/cncr.26392 22006014

[23] Bechis SK Carroll PR Cooperberg MR Impact of age at diagnosis on prostate cancer treatment and survival. J Clin Oncol (2011) 29:235–41 10.1200/JCO.2010.30.2075 21135285 PMC3058279

[24] Delongchamps NB Wang CY Chandan Y Jones RF Threatte G Jumbelic M Pathological characteristics of prostate cancer in elderly men. J Urol (2009) 182:927–30 10.1016/j.juro.2009.05.018 19616228

[25] Southwick PC Catalona WJ Partin AW Slawin KM Brawer MK Flanigan RC Prediction of post-radical prostatectomy pathological outcome for stage T1c prostate cancer with percent free prostate specific antigen: a prospective multicenter clinical trial. J Urol (1999) 162:1346–51 10.1016/S0022-5347(05)68282-1 10492194

[26] Wells TS Bukowinski AT Smith TC Smith B Dennis LK Chu LK Racial differences in prostate cancer risk remain among US servicemen with equal access to care. Prostate (2010) 70:727–34 10.1002/pros.21105 20033887

[27] Sanchez-Ortiz RF Troncoso P Babaian RJ Lloreta J Johnston DA Pettaway CA African-American men with nonpalpable prostate cancer exhibit greater tumor volume than matched white men. Cancer (2006) 107:75–82 10.1002/cncr.21954 16736511

[28] Amundadottir LT Sulem P Gudmundsson J Helgason A Baker A Agnarsson BA A common variant associated with prostate cancer in European and African populations. Nat Genet (2006) 38:652–8 10.1038/ng1808 16682969

[29] Gudmundsson J Sulem P Manolescu A Amundadottir LT Gudbjartsson D Helgason A Genome-wide association study identifies a second prostate cancer susceptibility variant at 8q24. Nat Genet (2007) 39:631–7 10.1038/ng1999 17401366

[30] Gaston KE Kim D Singh S Ford OH III Mohler JL Racial differences in androgen receptor protein expression in men with clinically localized prostate cancer. J Urol (2003) 170:990–3 10.1097/01.ju.0000079761.56154.e5 12913756

[31] Wallace TA Prueitt RL Yi M Howe TM Gillespie JW Yfantis HG Tumor immunobiological differences in prostate cancer between African-American and European-American men. Cancer Res (2008) 68:927–36 10.1158/0008-5472.CAN-07-2608 18245496

[32] Powell IJ Zhou J Sun Y Sakr WA Patel NP Heilbrun LK CYP3A4 genetic variant and disease-free survival among white and black men after radical prostatectomy. J Urol (2004) 172:1848–52 10.1097/01.ju.0000142779.76603.be 15540736

[33] Lane JA Hamdy FC Martin RM Turner EL Neal DE Donovan JL Latest results from the UK trials evaluating prostate cancer screening and treatment: the CAP and ProtecT studies. Eur J Cancer (2010) 46:3095–101 10.1016/j.ejca.2010.09.016 21047592

[34] Holmberg L Bill-Axelson A Helgesen F Salo JO Folmerz P Haggman M A randomized trial comparing radical prostatectomy with watchful waiting in early prostate cancer. N Engl J Med (2002) 347:781–9 10.1056/NEJMoa012794 12226148

[35] Bill-Axelson A Holmberg L Ruutu M Garmo H Stark JR Busch C Radical prostatectomy versus watchful waiting in early prostate cancer. N Engl J Med (2011) 364:1708–17 10.1056/NEJMoa1011967 21542742

[36] Wilt TJ Brawer MK Jones KM Barry MJ Aronson WJ Fox S Radical prostatectomy versus observation for localized prostate cancer. N Engl J Med (2012) 367:203–13 10.1056/NEJMoa1113162 22808955 PMC3429335

[37] Wright JL Salinas CA Lin DW Kolb S Koopmeiners J Feng Z Prostate cancer specific mortality and Gleason 7 disease differences in prostate cancer outcomes between cases with Gleason 4 + 3 and Gleason 3 + 4 tumors in a population based cohort. J Urol (2009) 182:2702–7 10.1016/j.juro.2009.08.026 19836772 PMC2828768

[38] Patel AA Chen MH Renshaw AA D’Amico AV PSA failure following definitive treatment of prostate cancer having biopsy Gleason score 7 with tertiary grade 5. JAMA (2007) 298:1533–8 10.1001/jama.298.13.1533 17911498

[39] Sim HG Telesca D Culp SH Ellis WJ Lange PH True LD Tertiary Gleason pattern 5 in Gleason 7 prostate cancer predicts pathological stage and biochemical recurrence. J Urol (2008) 179:1775–9 10.1016/j.juro.2008.01.016 18343432

[40] Whittemore DE Hick EJ Carter MR Moul JW Miranda-Sousa AJ Sexton WJ Significance of tertiary Gleason pattern 5 in Gleason score 7 radical prostatectomy specimens. J Urol (2008) 179:516–22 10.1016/j.juro.2007.09.085 18076949

[41] Servoll E Saeter T Vlatkovic L Lund T Nesland J Waaler G Impact of a tertiary Gleason pattern 4 or 5 on clinical failure and mortality after radical prostatectomy for clinically localised prostate cancer. BJU Int (2012) 109:1489–94 10.1111/j.1464-410X.2011.10583.x 21933333

[42] Lee AK Schultz D Renshaw AA Richie JP D’Amico AV Clinical utility of the percentage of positive prostate biopsies in defining biochemical outcome after radical prostatectomy for patients with clinically localized prostate cancer. Int J Radiat Oncol Biol Phys (2001) 49:673–7 10.1016/S0360-3016(00)01421-8 10715284

[43] D’Amico AV Schultz D Silver B Henry L Hurwitz M Kaplan I The clinical utility of the percent of positive prostate biopsies in predicting biochemical outcome following external-beam radiation therapy for patients with clinically localized prostate cancer. Int J Radiat Oncol Biol Phys (2001) 49:679–84 10.1016/S0360-3016(00)01423-1 11172949

[44] Grossfeld GD Latini DM Lubeck DP Broering JM Li YP Mehta SS Predicting disease recurrence in intermediate and high-risk patients undergoing radical prostatectomy using percent positive biopsies: results from CaPSURE. Urology (2002) 59:560–5 10.1016/S0090-4295(01)01658-2 11927314

[45] D’Amico AV Keshaviah A Manola J Cote K Loffredo M Iskrzytzky O Clinical utility of the percentage of positive prostate biopsies in predicting prostate cancer-specific and overall survival after radiotherapy for patients with localized prostate cancer. Int J Radiat Oncol Biol Phys (2002) 53:581–7 10.1016/S0360-3016(02)02797-9 12062600

[46] Freedland SJ Aronson WJ Csathy GS Kane CJ Amling CL Presti JC Jr Comparison of percentage of total prostate needle biopsy tissue with cancer to percentage of cores with cancer for predicting PSA recurrence after radical prostatectomy: results from the SEARCH database. Urology (2003) 61:742–7 10.1016/S0090-4295(02)02525-6 12670558

[47] Greene KL Elkin EP Karapetian A Duchane J Carroll PR Kane CJ Prostate biopsy tumor extent but not location predicts recurrence after radical prostatectomy: results from CaPSURE. J Urol (2006) 175:125–9 10.1016/S0022-5347(05)00056-X 16406887

[48] D’Amico AV Wu Y Chen MH Nash M Renshaw AA Richie JP Perineural invasion as a predictor of biochemical outcome following radical prostatectomy for select men with clinically localized prostate cancer. J Urol (2001) 165:126–9 10.1097/00005392-200101000-00031 11125380

